# Outcome Measurement in Economic Evaluations of Public Health Interventions: a Role for the Capability Approach?

**DOI:** 10.3390/ijerph7052274

**Published:** 2010-05-06

**Authors:** Paula K. Lorgelly, Kenny D. Lawson, Elisabeth A.L. Fenwick, Andrew H. Briggs

**Affiliations:** 1 Centre for Health Economics, Building 75, Monash University, Clayton 3800, Victoria, Australia; 2 Section of Public Health and Health Policy, 1 Lilybank Gardens, University of Glasgow, Glasgow, G12 8RZ, UK; E-Mails: k.lawson@clinmed.gla.ac.uk (K.D.L.); e.fenwick@clinmed.gla.ac.uk (E.A.L.F.); a.briggs@clinmed.gla.ac.uk (A.H.B.)

**Keywords:** economic evaluation, outcome measures, public health, capability approach

## Abstract

Public health interventions have received increased attention from policy makers, and there has been a corresponding increase in the number of economic evaluations within the domain of public health. However, methods to evaluate public health interventions are less well established than those for medical interventions. Focusing on health as an outcome measure is likely to underestimate the impact of many public health interventions. This paper provides a review of outcome measures in public health; and describes the benefits of using the capability approach as a means to developing an all encompassing outcome measure.

## 1. Introduction

Public health interventions are intended to promote health or prevent ill health in communities or populations, and can be distinguished from clinical or medical interventions which intend to prevent or treat ill health in individuals [1]. The nature of public health interventions and programmes has evolved considerably over time. This evolution has been summarized by Eriksson as four generations or paradigms [2]: single factor interventions have given way to multifactorial interventions which then became community based, and now we have nearly come full circle with a returned focus on policy and environmental actions; from the ‘old public health’ to the ‘new public health’ [2,3]. As a consequence interventions are becoming more complex [4], where the complexity lies in the intervention, the outcomes and the evaluation itself.

Limited budgets and competing demands have resulted in a growing need for economic evidence to guide decision making regarding the funding of both health (medical) technologies and public health programmes. A review of the literature from 1966 to 2005 has documented the growth in economic evaluations of public health interventions; only 12% of nearly 1,700 papers pre-dated 1999 [5]. The review found that the majority of papers were concerned with evaluating the prevention of communicable diseases (60%), or the evaluation of screening or diagnostic tools for cancer (35%); and 78% of all papers undertook cost effectiveness analyses (CEA) or cost consequence analyses (CCA) (see Simoens [6] in this special issue for a health economics primer which reviews the different types of economic evaluation approaches). The importance of evaluating public health programmes has been noted at a policy level in the UK. The Wanless Reports [7,8] suggested that an efficient approach to improving the health of the population and reducing health inequalities includes the generation of evidence on the cost-effectiveness of public health strategies. As a result of these recommendations the National Institute for Clinical Excellence (NICE) subsumed the role of the Health Development Agency (HDA) and now the National Institute for Health and Clinical Excellence (NICE) is tasked with providing guidance on the effectiveness and cost effectiveness of public health interventions in the UK.

While Wanless argued that the “[e]conomic evaluation of public health interventions is not inherently different from the evaluation of other health interventions. Standard principles are the same” [8, p. 146], there are in fact a wealth of complexities in the application of the methodology that need to be addressed before economic evaluations of public health interventions are able to produce quality evidence to inform rationing decisions; especially when the decision makers are comparing different types of public health programmes, and comparing public health interventions with other health care interventions. This paper initially reviews the methodological challenges that have been previously identified [9,10], and then specifically focuses on the issue of the measurement of outcomes. A methodological discussion of outcome measurement is presented, which includes a selective review of a number of recently published economic evaluations of public health interventions. The paper then focuses on operationalising Sen’s [11,12] capability approach as a means of measuring benefit, before concluding with a discussion of a number of the outstanding issues, and avenues for future research.

## 2. Methodological Issues in the Economic Evaluation of Public Health Interventions

The problems of applying economic evaluation to public health interventions were first outlined in an HDA briefing paper [13]. This highlighted the need for a common framework for consistent and transparent decision making, which was flexible enough to capture the multi-dimensional, complex and layered outcomes of public health policies and interventions. These issues and others were further explored in a paper co-authored by members of NICE, the so-called decision-makers [9]. They detailed the challenges of producing public health guidance under NICE’s expanded remit. Seven issues were identified which were labeled as research priorities. These include:

– *Measuring benefit*, the use of quality adjusted life years (QALYs) (and EQ-5D) and the possible need for evaluations to have more than one outcome measure;
– *Public versus individual*, the role of individual choice in population based interventions, and how to account for any resulting externalities;
– *Equity versus efficiency*, public health programmes frequently target health inequalities, such that the issue of weighting outcomes may need to be addressed together with other distributional concerns;
– *Perspective*, in the NICE Reference Case [14] the perspective for public health evaluations has been broadened to include the public sector, this may lead to inconsistencies when making comparisons with clinical interventions;
– *Extrapolation*, what is the appropriate time horizon and how meaningful will such extrapolations be in the absence of robust evidence;
– *Quality of evidence*, the evidence base is weaker in public health, and controlled trials are often impossible;
– *Cost effectiveness threshold*, should the same threshold be applied to both clinical and public health interventions.

A more rigorous assessment of the issues of applying standard economic analysis techniques to public health evaluations has also been undertaken [10]. Weatherly and colleagues considered existing reviews of the literature, which included the Wanless Reports [7,8], and identified what they regard to be the main methodological challenges facing health economists in this area. These include:

– *Attribution of outcomes*, how best to obtain true estimates of effect, what can the existing literature offer by way of evidence, how can primary research generate quality evidence, and what is the appropriate time frame within which to measure success;
– *Measuring and valuing outcomes*, what can be measured *versus* what should be measured, the need for a more generic measure of wellbeing, and sector-specific generic measures of outcome, as well as greater consideration for alternative evaluation approaches;
– *Intersectoral costs and consequences*, quantifying the intersectoral impacts of public health interventions, assessment of a general equilibrium approach to the evaluation of public health interventions;
– *Equity considerations*, a need for heath inequality impact assessment and research on equity weighting.

Given these four areas they then undertook a review of the empirical literature in eleven public health domains (accidents, alcohol, ante natal and post natal visiting, drug use, HIV/AIDS, low birth weight, obesity and physical activity, sexually transmitted infections, smoking, teenage pregnancy and youth suicide prevention) and concluded that the published literature offers few insights and there is little in the way of best practice, suggesting more methodological research is required.

In the rest of this paper we wish to focus on a single issue identified by both Chalkidou *et al.* [9] and Weatherly *et al.* [10] that of the measurement (and valuation) of benefits (outcomes). A number of the other issues have already been addressed in the literature, including work on systematic review methods [15], the implications of alternative perspectives [16,17], and the incorporation of equity considerations [18], as well as more general guidance on how to evaluate complex interventions [19,20].

## 3. Outcome Measurement in Economic Evaluation

Economists are often merely seen as experts on costing, but outcome measurement is a key issue in economic evaluation [21]. Outcome measures used in economic evaluations can generally be categorized as one of the following:

– *Condition specific*, for example episode free days, which would have different meanings for say asthma [22] and gastroesphageal reflux disease [23];
– *Morbidity*, clinical measures of, say, prevalence or events [24], generally expressed in natural units;
– *Generic health or quality of life*, such as the SF-36 [25] or Sickness Impact Profile [26];
– *Mortality*, so to estimate life years gained;
– *Preference based*, either generic like the EQ-5D [27] or SF-6D [28] or condition specific [29,30], which allow for the estimation of QALYs;
– *Monetary*, as measured in a contingent valuation exercise to elicit an individual’s willingness-to-pay for an intervention [31].

The choice of outcome measure is very dependent on the research question being addressed (which includes the perspective employed) and the type of economic evaluation that is being undertaken. A CEA, where outcomes are expressed in natural units, remains a relatively common approach within health technology assessment, and this is also true for public health interventions. McDaid and Needle [5] found in their review that 57% of all published evaluations were CEAs, while Weatherly *et al.* [10] found in their more selective review that 36% of studies were CEAs. Some examples of published CEAs of public health interventions include evaluations of: targeted screening for cardiovascular disease which estimate the cost per case [32]; vaccination programmes which estimate the cost per hospitalization avoided [33]; surgical interventions for obesity which estimate the cost per pound lost [34]; and behavioral interventions for smoking cessation which estimate the cost per quitter [35]. An example of a more complex public health intervention is the recently published evaluation of an intervention for vulnerable families [36]. Here the authors estimated the cost per improvement in maternal sensitivity and cost per improvement in infant cooperativeness (components of the CARE Index).

While CEA are commonplace they are limited in that they can only inform decisions within individual disease or intervention areas. In order to facilitate comparisons across a range of topics, diseases and interventions, including both life saving and life enhancing interventions, a common generic outcome measure which incorporates the effects of both quality and quantity of life was developed. A QALY combines both mortality and morbidity measures of health by weighting a year of life by the quality of life (that is utility) experienced [37]. This quality adjustment explicitly involves an expression of preference which can be elicited by employing a range of preference elicitation techniques (like time-trade off or standard gamble [38]), but generally off the shelf instruments are used like the EQ-5D (a five dimension questionnaire) [27], or the more recent utility values extracted from the SF-36, using the SF-6D [39]. Once estimated QALYs are compared to costs in the form of an incremental cost effectiveness ratio (ICER) and comparisons across interventions and disease areas can be made using cost per QALY gained, thereby informing decisions as to whether an intervention can be considered value-for-money.

Some examples of published cost utility analyses (CUAs) of public health interventions include those that estimate the cost per QALY gained for diabetes screening [40], vaccination programmes [41], surgical interventions for obesity [42], and smoking cessation [35]. There are also CUAs that employ disability adjusted life years (DALYs) as an outcome measure, thereby estimating the cost per DALY saved [43], averted [44] or recovered [45].

QALYs (and suggested alternatives, such as the healthy year equivalent (HYEs) [46] and the saved young life equivalent (SAVE) [47]) are, however, not without their critics. As discussed above, one of the limitations is that they focus on health outcomes [48,49], and there is now a need to evaluate interventions that seek to improve an individual’s quality of life beyond health. Many public health interventions seek to impact on broader aspects of quality of life, not just health, but also non-health outcomes such as empowerment, participation and crime. Therefore, QALYs and their associated quality of life measures like the EQ-5D or SF-6D are likely to underestimate the relative benefits of public health interventions when compared to health care interventions.

An alternative approach to valuing outcomes, which can potentially overcome this bias, and capture all benefits (both health and non-health) of interest is the contingent valuation method. Contingent valuation (CV) is a means by which outcomes are valued in monetary terms. The most common approach to eliciting monetary valuations is to use the willingness-to-pay (WTP) approach [31]. In its simplest form, individuals are asked how much they would be willing to pay to obtain the benefit of an intervention. If this monetary valuation is greater than the cost of providing the intervention, then a cost-benefit analysis (CBA) would suggest that the intervention is worthwhile. There are a number of practical and methodological problems with the CV approach [50], in particular there is a strong relationship between income and WTP, whereby those on low income provide low valuations. In the context of evaluating public health intervention this could be problematic, as many interventions are targeted at deprived individuals, such that the use of WTP could undervalue the true benefit. While, Kelly *et al.* [13] conclude that, at a societal level, CBA is the ideal method, as it permits tradeoffs across different sectors of the economy, they admit that there are problems with this approach, and they go on to suggest that within a pragmatic framework cost-consequence analysis (CCA) may be able to capture the layered outcomes of public health interventions. Note that few real world examples of CBA exist, indeed of the four studies that were initially identified as CBA in the review by Weatherley *et al.* [10] three were subsequently deemed to be CCAs and one was a CUA; although they did identify one study which elicited WTP values for a water fluoridation programme, but it did not estimate the costs of the programme [51].

A CCA [52], unlike the approaches described above, does not explicitly compare the costs of an intervention with its outcomes (thus is not an economic evaluation in the strict sense). Multiple outcomes are presented, often in a tabulated approach with costs, and while it cannot be used to rank interventions, it has been heralded as a better way to present (often confusing) economic information to decision makers [53]. CCA has been used previously to evaluate complex interventions where outcomes cannot easily be summarized in a single measure (see Byford and Sefton [54] for some examples).

Sen’s capability approach [11,12] could provide a possible solution to the limitations discussed above, in that it expands the evaluation space to consider whether a programme enhances an individual’s capability. While there is much (theoretical) discussion of the application of the ‘capability approach’ within the health economics (including economic evaluation) literature, there are few applications of the approach. We first review the approach as put forward by Sen and his supporters, before providing a discussion of the theoretical literature within the health economics/economic evaluation domain. We then provide a short discussion of the applied approaches to measuring so-called capability sets.

## 4. The Capability Approach

The capability approach, as put forward by Sen [11,12], suggests that wellbeing should be measured not according to what individuals actually do (functionings) but what they can do (capabilities).

“*Functionings* represent parts of the state of a person—in particular the various things that he or she manages to do or be in leading a life. The *capability* of a person reflects the alternative combinations of functionings the person can achieve, and from which he or she can choose one collection. The approach is based on a view of living as a combination of various ‘doings and beings’, with quality of life to be assessed in terms of the capability to achieve valuable functionings.” [12, p. 31]

Comim neatly described the approach as “a framework for evaluating and assessing social arrangements, standards of living, inequality, poverty, justice, quality of life or wellbeing” [55, p. 162]. Of importance is the evaluation space; it diverges from narrow utility space, which is concerned with the pleasure obtained from the consumption on goods and services, and instead encapsulates an informational space, where evaluative judgments occur according to an individual’s freedom. Therefore, Sen’s approach is based on value judgments, which ultimately relate to an individual’s capability set, and in this sense it can be described as ‘extra-welfarist’ [56–58].

The capability framework for evaluation is based on two distinctions, that between a person’s agency goals and their own wellbeing (where agency goals refer to the notion that individuals may have objectives which relate to the well-being of others and to commitments entirely outside themselves [59]), and that between achievement (functioning) and the freedom to achieve (capabilities). Arguably one of the limitations of the approach is that “Sen has not specified how the various value judgments that inhere in his approach and are required in order for its practical use (whether at the micro or macro level) are to be made” [60, p. 3], as he believes that value selection and discrimination are an intrinsic part of the approach. Nussbaum [61], however, has identified what she regards as central human capabilities, and provides a list of ten capabilities: life; bodily health; bodily integrity; senses, imagination and thought; emotions; practical reason; affiliation; other species; play; and control over one’s environment. Other prescriptive lists also exist, which have varying degrees of abstraction and generalization [62]. The existence of such lists are crucial in the evaluation of capability sets (that is the identification of freedoms) and the subsequent operationalisation of the approach (that is evaluating whether such freedoms are achievable).

### 4.1. The Application of the Approach to Health Economics—Theoretical Literature

The first insight to the significance that the capability approach might have within the health economics domain became apparent when Culyer [56] used Sen’s theory to develop his own extra-welfarist perspective to economic evaluation (which provided some justification for using QALYs). This perspective, as discussed above, is limited in that it focuses on health, while Sen’s capability approach is much broader. Furthermore, Culyer’s approach is largely concerned with functionings (the achievement of health states) compared with Sen’s ideas on the ability to function [63].

Anand has advanced the approach, first discussing the application of the approach to health care rationing and resource allocation [64,65] (including editing a special issue in *Social Science and Medicine* [66]) and more recently by attempting to operationalise the approach [67,68]. However, it was Cookson [69] who first explored the possibility of applying the approach to outcome measurement within economic evaluation. He suggests that there are three ways it could be used:

- direct estimation and valuation of capability sets;
- ‘merging’ preference-based measurements, such as willingness to pay, with capabilities;
- re-interpreting the QALY approach.

Cookson dismisses the first approach as unfeasible at present, arguing that there is no agreed list of functionings, and that any movement from functionings to capabilities is problematic due to different preferences. While the second approach is also dismissed due to “the adaptive and constructed nature of individual preferences over time and under uncertainty” [69, p. 818]. Subsequently, Cookson proposes re-interpreting QALY data generated from a standardised instrument so that the re-interpreted data (the ‘capability QALY’, note others refer to this all encompassing concept as the ‘super QALY’) represents the value of an individual’s capability set. He argues that responses to questions in generic health state valuation instruments can be taken to reflect the value of an unspecified capability set, because health affects an individual’s freedom to choose non-health activities.

Anand [70] disputes Cookson’s conjecture that capability measurement is not yet feasible. In particular he claims while early attempts to measure capability concluded that it was immeasurable, it is now much more feasible to measure capability (indeed the UN’s Human Development Index has it’s foundations within the capability approach). Anand identifies Nussbaum’s list of ten domains as a good starting point, and then shows that many of these are well represented by questions in the British Household Panel Survey (BHPS), a large longitudinal survey extensively used by economists and social scientists alike [67,68].

Recently Coast *et al.* [63,71] have sought to reignite the debate surrounding the application of the capability approach within health economics. Whilst tracing the origins and impacts of extra-welfarism on health care policy, they discuss a number of the issues surrounding further integration and application of the approach for use in economic evaluation. They highlight the issue identified above, that the capability approach has a wider evaluative space, but also focus on the fact that extra-welfarist approaches seek to maximize health, whereas the capability approach is more concerned with issues of equity, distribution, and the equality of basic capabilities [72]. Thus, while we are presenting the capability approach as a means to overcoming the problems associated with measuring outcomes for public health, the approach could also provide a solution to addressing a number of the equity issues that have been raised [9,10].

### 4.2. The Application of the Approach to (Health) Economics—Empirical Literature

The literature on capabilities, whilst extensive, remains largely conceptual. Robeyns in a review of the literature in 2000 noted that “despite the fact that Sen published Commodities and Capabilities in 1985, the number of empirical applications is still quite limited” [73, p. 26] (see Kyklys and Robeyns [74] and Comim *et al.* [75] for more up-to-date reviews). Despite this there have been some empirical applications, the majority of which relate to poverty, development, social justice or gender inequality (see [74,76]), although there are a (growing) number in the health economics field.

As discussed above, Anand has sought to operationalise the approach by assessing capabilities using secondary data. He (and colleagues) exploited data from the BHPS and estimated the relationship between wellbeing and capability [67]. They concluded that secondary data sources can provide *some* information on capability. The incompleteness led them to consider other data sources and they subsequently developed further indicators, which are aligned within Nussbaum’s list of ten capabilities [61]. These indicators were included in an internet survey, along with measures of wellbeing, and the indicators of capability were found to perform well in terms of being strong predictors of wellbeing [68]. The drawback of their approach, however, in terms of outcome measurement for economic evaluation is that there are over 60 indicators of capability, making its usability limited.

Further research sought to reduce and refine Anand’s survey, so to provide a summary measure of capability which could be used when evaluating complex public health intervention [77]. The reduction and refinement of the questionnaire took place across a number of stages, using both qualitative (focus group discussions and in-depth interviews) and quantitative (secondary data analysis and primary data collection using postal surveys) approaches [78]. The final stage tested the validity of the questionnaire. The questionnaire was reduced from its original 65 questions to 18 specific capability items, which remain aligned with Nussbaum’s list of central human capabilities. The finalised questionnaire, and a weighted index of capability (whereby each item was given the same weight), was found to be responsive to different groups of individuals (as categorised by age, gender and deprivation), and measure something additional to health and wellbeing (as measured by the EQ-5D and a global QoL scale, respectively), although was still highly correlated with these measures. This research shows the potential to operationalise the capability approach, despite Cookson’s reservations [69]. However, one drawback was that a preference based index was not developed (that is one that represents trade-offs and choices across the capability set), as it was deemed beyond the scope of the project; this is however wholly possible, and other researchers have successfully developed a preference based index.

Coast and colleagues have developed an index of capability specifically for use in the elderly [79–81]. While eliciting attributes for a generic quality of life measure for older people (by interpreting in-depth qualitative interviews), a similarity became apparent between the resulting attributes (attachment, role, enjoyment, security and control) and Sen’s capability approach. The attributes were valued using best-worst scaling within a discrete choice framework [82]; and were combined to form an index, whereby 0 represents a state of no capability and 1 is a state of full capability. Their approach has many merits, especially their choice of valuation technique, but it is limited in its generalisability beyond the elderly. The research team has since been funded to undertake a similar exercise with a broader scope, and they are now seeking to measure capabilities in the general adult population [83].

Other health economists have used the approach to assess the quality of life of sufferers of chronic pain [84]. This project used a multi-attribute value method [85] to scale the levels within functionings and quantify trade-offs between capabilities [86]. Within the broader area of health (but not specifically health economics), there have been a number of papers which have also attempted to estimate capability. In particular, disability appears to readily lend itself to the capability approach [87], and there have been attempts to estimate the additional income needed by a disabled person to reach the wellbeing of a non-disabled person [88,89].

### 4.3. Outstanding Issues With Regard to Operationalising the Approach for Use in Economic Evaluations

A fundamental issue with operationalising the capability approach for use in economic evaluations is the need to develop a preference based measure, such that it reflects the relative value placed on the various dimensions and components of capability. However, the method by which values should be elicited remains unclear [80]. Cookson [69] dismissed the valuation of capability sets as unfeasible, citing Sen [11,90] who rejects the use of either choices or desires to value capabilities, and instead suggested that perhaps views on value judgements be elicited instead. Coast and colleagues [80] argue that their best-worst scaling approach, because respondents are asked to only specify the attribute levels which they think are the best and worst, elicits ‘values’ (as Cookson suggests) rather than ‘choices’, because the elicitation exercise does not ask individuals to risk or sacrifice, as would be the case in a standard gamble or time trade-off exercise, respectively.

There has been some research comparing cardinal valuation methods which elicit the degree of preference (as is the case with standard gamble, time tradeoff and visual analogue scale methods) to ordinal methods which elicit information on the ordering of preference using conventional Discrete Choice Experiment (DCE) models [91] and best-worst scaling approaches [92]. DCEs were found to have a number of practical advantages, including the fact that less abstract reasoning is required on the part of the respondents (compared to time tradeoff and standard gamble exercises); but due to their ordinal nature the elicited values require rescaling, that is they need to be anchored. To produce a measure which can be used to weight length of life (as is the case with QALYs) [93] and allow for interpersonal comparisons [94], it is necessary that the scale is anchored at zero which is dead, with full health being one. While Ratcliffe and colleagues [91] show that it is possible to undertake such rescaling using rank and DCE data for a condition-specific health measure, within the context of the capability approach there is a further unresolved issue. Although it is generally accepted that the absence of health is the same as the absence of life (that is it is appropriate that dead be given a value of zero), there has been little debate about whether the absence of capability is the same as the absence of life; but if a capability index is to be used in a similar manner to a QALY such a discussion is required. Notably, Coast *et al.* [80] take a philosophical approach where the absence of capability is given a value of zero, thereby avoiding the need to value death.

Adaptation, where individuals may not recognise their own lack of wellbeing because they have adapted to their situation, is also an issue when undertaking a preference based valuation. This is despite the fact that Sen used the issue of adaptation as the basis for, rejecting utilitarian approaches which seek to value wellbeing and, replacing utility with an informational space based on functionings [90]. Adaptation is an issue when using the public to value hypothetical health states as the valuation often reflects the initial shock response, rather than the long term (patient) experience; this means that the general public often give lower values compared to patients who may have adapted [95]. The measurement of preferences for different capability states could also suffer from similar adaptation issues (with the additional issue of functioning in one domain potentially over compensating for a lack of capability in another). Burchardt [59] has shown that agency goals are adaptive, and that an assessment of inequality based on agency goals may be bias because of lower aspirations (when setting goals), and therefore greater success in achieving goals. One solution to adaptation that Sen has advocated is using an expert-centred approach; such that in the public health context, public health professionals or policy makers would be used to provide values for different capability states [71]. This is entirely plausible but conflicts with the current movement towards patient and public involvement in decision making.

## 5. Conclusion

The need to undertake economic evaluations across a wider range of interventions, which encompass both health and non-health outcomes, requires an alternative to the conventional cost per QALY gained approach. Sen’s capability approach, although theoretically challenging, could provide a possible solution.

The benefits of using the capability approach are numerous. It offers a much richer set of dimensions for evaluation, which given the nature of public health and social interventions, with their many and complex outcomes, makes the approach ideal for capturing all these outcomes, rather than focusing solely on health status. The equitable underpinnings of the approach are also appropriate for use with public health interventions which often involve reducing inequalities across groups (namely improving deprivation) as an overriding aim.

To operationalise the approach for use in economic evaluations, it is necessary to generate an index whereby an individual’s capability (or capability set) is described by a single composite number. This involves a number of challenges. Key among these is the need to identify a legitimate capability space and then to accurately measure relative preferences for each capability. Indices, and preference measurement more generally, raise the issues of which valuation technique to use, whether and how to anchor the index, and how to control for adaptation.

Future research will contribute to the removal of many of the conceptual challenges; however, in the long run a potential institutional barrier to the adoption of a capability approach is that the QALY-based extra-welfarist approach is now the norm in health economics. For instance, within the UK NICE has a clear recommendation that QALYs should be used as the reference case, and research on methods for cost effectiveness analysis (as opposed to outcomes research) continues to grow. Although there are a number of alternative approaches (experienced utility [96] and happiness/life satisfaction/wellbeing [97,98]) which provide possible competition if support for the extra-welfarist approach was to waiver, the capability approach would appear to have strength as a means of measuring the effectiveness (and thus cost effectiveness) of public health interventions.
